# Exploring phytochemical composition, photocatalytic, antibacterial, and antifungal efficacies of Au NPs supported by *Cymbopogon flexuosus* essential oil

**DOI:** 10.1038/s41598-022-15899-9

**Published:** 2022-08-22

**Authors:** Diksha Pathania, Mamta Sharma, Pankaj Thakur, Vishal Chaudhary, Ajeet Kaushik, Hidemitsu Furukawa, Ajit Khosla

**Affiliations:** 1 School of Biological and Environmental Sciences, Shoolini University, Solan, Himachal Pradesh 173212 India; 2 Department of Botany, Sardar Patel University, Mandi, Himachal Pradesh 175001 India; 3 Special Center for Nanoscience, Jawaharlal Nehru University, New Delhi, 110067 India; 4 Research Cell and Department of Physics, Bhagini Nivedita College, University of Delhi, New Delhi, 110075 India; 5 NanoBio Tech Laboratory, Health System Engineering, Department of Environmental Engineering, Florida Polytechnic University, Lakeland, FL 33805-8531 USA; 6 School of Engineering, University of Petroleum and Energy Studies (UPES), Dehradun, Uttarakhand India; 7 Department of Mechanical Systems Engineering, Graduate School of Science and Engineering, Yamagata University, Yonezawa, Yamagata 992-8510 Japan; 8 School of Advanced Materials and Nanotechnology, Xidian University, Xi’an, 710126 People’s Republic of China

**Keywords:** Biochemistry, Green chemistry

## Abstract

Biofabrication of gold nanoparticles (AuNPs) using the aromatic essential oils is highlighted due to its simple, economical, low toxicity, and eco-friendly nature. Essential oil of *Cymbopogon flexuosus* (*CF*), an economically valuable medicinal plant, exhibits anti-inflammatory, anti-tumor, antioxidant, and antimicrobial activities. For the first time, this research accounts for the biosynthesis, physicochemical, photocatalytic, antifungal, antibacterial properties of biogenic AuNPs, fabricated using CF essential oil collected from different altitudes (S1-Palampur, S2-Haryana, S3-Dehradun). The altitudinal disparity in the phytochemical composition of essential oils is highlighted. The average crystallite size ranged from 10 to 32 nm and was influenced by CF samples used in the synthesis. The spectroscopic outcomes revealed the involvement of bioactive reagents from CF essential oil in the fabrication and stabilization of AuNPs. The fabricated AuNPs exhibited excellent antimicrobial activities against all tested strains (*Staphyloccucs aureus*, *Escherichia coli, Fusarium oxysporum*), showing their efficacy as an antimicrobial agent to treat infectious diseases. Moreover, AuNPs exhibited excellent photocatalytic efficacy of around 91.8% for the degradation of methylene blue under exposure of direct sunlight for 3 h without the assistance of an external reducing agent. The outcomes highlight a potential economic and environmentally friendly strategy to fabricate biogenic AuNPs for diversified industrial applications where antimicrobial and photocatalytic efficacies are the key requirements.

## Introduction

The altitudinal variation is one of the intriguing elements, which may aid in understanding the medicinal effects of diverse plant species. Biofabrication of secondary metabolites in medicinal plants is affected by biotic and abiotic stresses in addition to genetic regulation^1^. Plants perform differently in provisions of biochemical elements in distinct ecological niches to better acclimatize to their surroundings. This diverse set of ecological conditions varies in altitude, affecting the chemical makeup and, eventually, the endurance of therapeutic plants in these areas. Plants secondary metabolites or chemicals, which are usually the basis for their therapeutic efficacy, are affected by environmental factors^2,3^. At a more significant elevation, plant variation is structured by several physiological and biochemical mechanisms that avoid and overcome stress circumstances. Production of oxidative stress, bioactive compounds, and the progression of resistance-inducing genotypes that create environmentally suited phenotypes are among the mechanisms mentioned^4^.

In this decade, nanotechnology has been a prominent field in the research area. Researchers and scientists hope that the continued advancement of nanotechnology will lead to improvement in quality of life^5,6^. Recent developments in the area of nanotechnology, as like the capacity to create highly well-organized nanoparticles of every structure and size, have controlled the discovery of novel biocidal mediators^7^. Metallic nanocomposites have been increasingly employed in current years because of their distinctive visual, electrical, alluring, chemical, and mechanical characteristics, the possessions of which are vastly different from those of mass substances^8,9^. Their small size and substantial surface areas might be attributable to these distinct and unique characteristics, but there is still much to learn about their environmental influences^10,11^. When it comes to metal nanoparticles, AuNPs have received tremendous interest because of biosensors, biomedicine, pharmaceutical, electrical conductivity, optical, and catalysis applications in the last few years^12–17^. The following procedures were used to generate gold nanoparticles with regulated shape and size in the early stages of research which are: (a) photo-reduction, (b) ultrasonic radiation, (c) microwave irradiation, (d) thermal decomposition in the organic solvent, (e) chemical reduction of gold ions, and (f) reversed micelle process. Nevertheless, in most situations, these approaches pose health and environmental risks^18,19^.

In the past several years, there has been an increasing necessity to create ecologically sustainable nanoparticles synthesis strategies, amongst which green synthesis of nanoparticles has garnered significant attention^20^. It is also essential to have a simple setup for a reaction, modest reaction circumstances, the use of safe solvents like water, the removal of potentially hazardous and harmful components, and low costs. This procedure is compatible with biomedical and pharmaceutical applications as well^21^. This approach does not consult for increased pressure, power generation, or potentially hazardous chemicals^22^. Studies on the green production of AuNPs, including plant extract, microorganisms, and biopolymers are many. Even with the accessibility of technologies for the biofabrication of AuNPs by diverse plant extract, the probable of plants as organic resources for the formation of nanoparticles must be investigated^23^.

Clarance et al. fabricated gold nanoparticles with an endophytic strain *Fusarium solani*, isolated from *Chonemorpha fragrans* by green fabrication method, and anticancer activity was investigated. The development of AuNPs was investigated through UV–visible analysis, XRD, FTIR, and SEM analysis^24^. Rokade et al*.* fabricated gold nanoparticles through novel green synthesis for the catalytic degradation of 4-nitrophenol. Fabricated AuNPs were confirmed through UV–visible, XRD, DLS, EDX, and TEM analysis^25^. Lemongrass (*Cymbopogon flexuosus*) is a class belonging to the Poaceae (grass) family. It is grown in subtropical and tropical zones of Asia and around the world is a significant producer. Lemongrass essential oil contains several constituents: citronellal, limonene, geranyl acetate, citral, geraniol, neral, and others^26,27^. The high amount of citral constituents, limonene, and geranyl acetate are responsible for antimicrobial, anticancer, anti-inflammatory, and analgesic characteristics^27,28^. There have also been a few studies that show that lemongrass essential oil can be utilized as a biofuel, due to this, lemongrass is considered an energy plant^29,30^. These characteristics make lemongrass a highly desirable crop for the pharmaceutical, cosmetics, food, and perfume industries due to its great potential in these fields^31^. Saada et al*.* investigated the lemongrass essential oil for antibacterial efficacy against four different bacterial strains in its ordinary and nano-form. A low-energy emulsification technique was used to form an oil nanoemulsion^32^. S. Aswathy Aromal and Daizy Philip fabricated Au nanoparticles utilizing an aqueous extract of *Trigonella foenum*-*graecum* through green synthesis intended to reduce 4-nitrophenol to 4-aminophenol by surplus NaBH_4_^33^. Similarly, Smitha et al*.* synthesized Au nanoparticles by *Cinnamomum zeylanicum* leaf broth through green synthesis^34^. Likewise, Philip et al*.* fabricated AuNPs using leaf extract of *Murraya Koenigii* by green synthesis^35^. Due to pitiable substantial properties such as susceptibility to degradation, hydrophobicity, and volatility, Lemongrass essential oil makes them complicated to be used in pharmaceutical implementations. To overcome this disadvantage to a level by encapsulating the essential oil into a nano-carrier, helpful in target discharge and prohibited release at ailing sites.

Considering above discussed developments into consideration, for the first time, this research investigates the physicochemical, antifungal, antibacterial, and photocatalytic characteristics of *Cymbopogon flexuosus* essential oil mediated AuNPs. Moreover, the influence of altitudinal variation on phytochemical compositions of derived essential oil and antimicrobial, photocatalytic, and physicochemical properties of fabricated AuNPs is highlighted using various spectroscopic investigations. The antibacterial and antifungal efficacies of fabricated biogenic AuNPs were evaluated against *Staphyloccucs aureus*, *Escherichia coli, Fusarium oxysporum*, respectively. The sunlight assisted photodegradation efficiency of AuNPs was evaluated against Methylene Blue (MB) dye.

## Results and discussion

### Compositional analysis and altitudinal disparity of derived *Cymbopogon flexuosus* essential oils

The phytochemical analysis of essential oil extracted from *Cymbopogon flexuosus* collected from different altitudes, i.e., Palampur (1385 m), Sahaspur [Dehradun] (684 m), and Khizrabad [Haryana] (245 m) is performed by GCMS technique. The plants showed a variation in the concentration of their volatile constituents affected by season and altitude^36^. It is observed that essential oils encompass citral and linalool occupy a prominent place in provisions of anti-inflammatory activity, caryophyllene that possesses anti-inflammatory, antimicrobial, and analgesic activity^37^. Various constituents present in essential oils like Citral, photocitral-B, caryophyllene oxide, α humulene, and cis-verbenol have been investigated to possess antimicrobial potency^38^.

### From site I—Palampur (S1)

**Figure 1:**
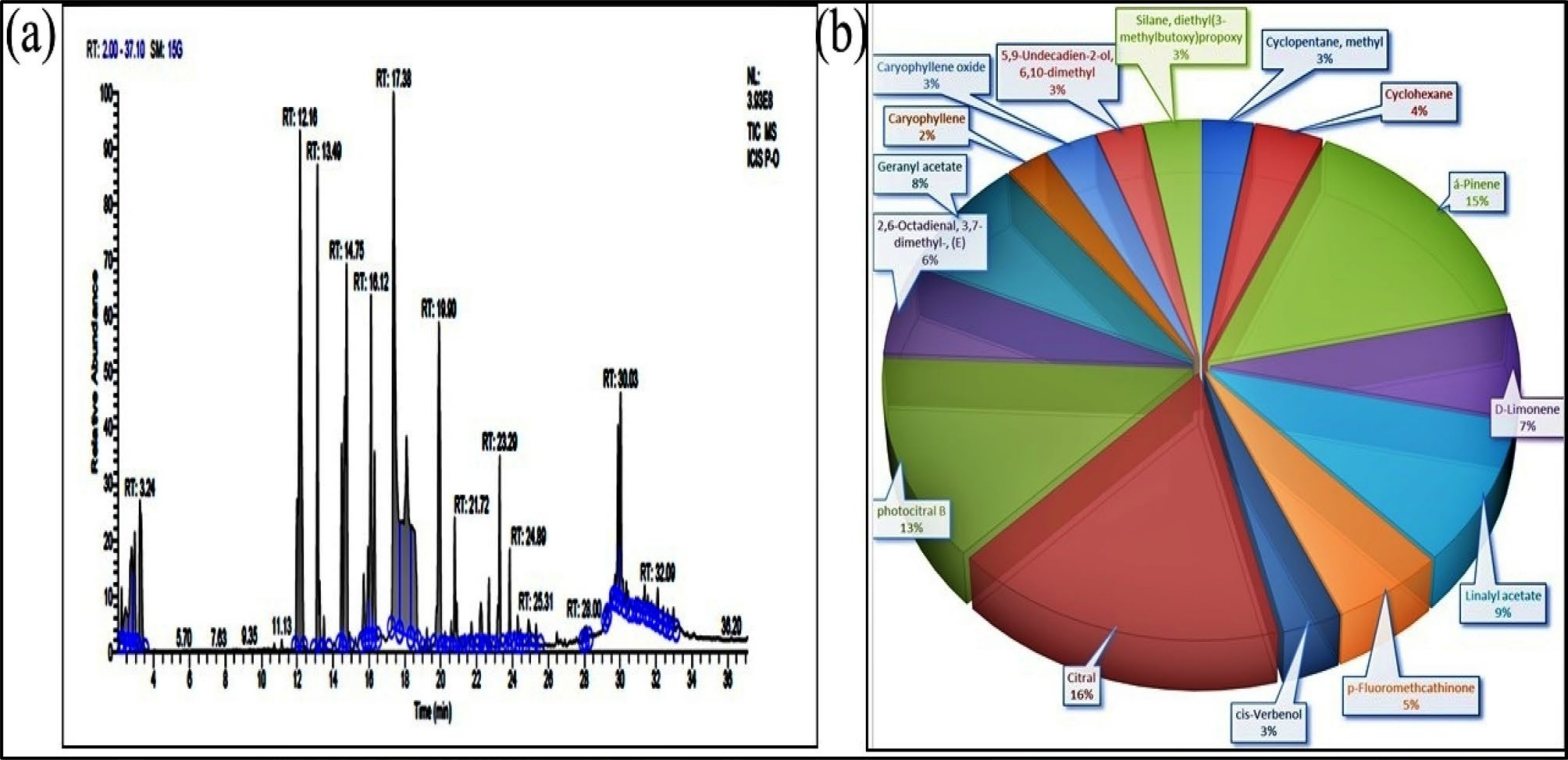
(**a**) Total ion chromatograms of GC–MS, (**b**) Percentage chemical composition of essential oil of *C. flexuosus* from site I—Palampur (S1).

In S1, chromatogram of essential oil of *C. flexuosus* were characterized with total 50 prominent peaks at different retention times, i.e., 2.21–32.97 Compounds Citral and Photocitral B observed at RT-17.38 with peak area 13.61% and RT-18.10 with peak area 10.79% respectively, and α- pinene with peak area 12.39% at RT-12.16 were major compounds. Other compounds observed were Geranyl acetate, linalyl acetate, D-limonene, p-Fluoromethcathinone, cyclohexane, Cis-Verbenol, Cyclopentane, Caryophyllene oxide (Fig. 1). Other minor compounds are Caryophyllene, Humulene, Epizonarene, Hinesol, Carotol, Ambrosin, Camphorsulfonic acid, Thunbergol and Dihydrojasmone, which were present in a minimal quantity.

### From site II—Haryana (S2)

**Figure 2:**
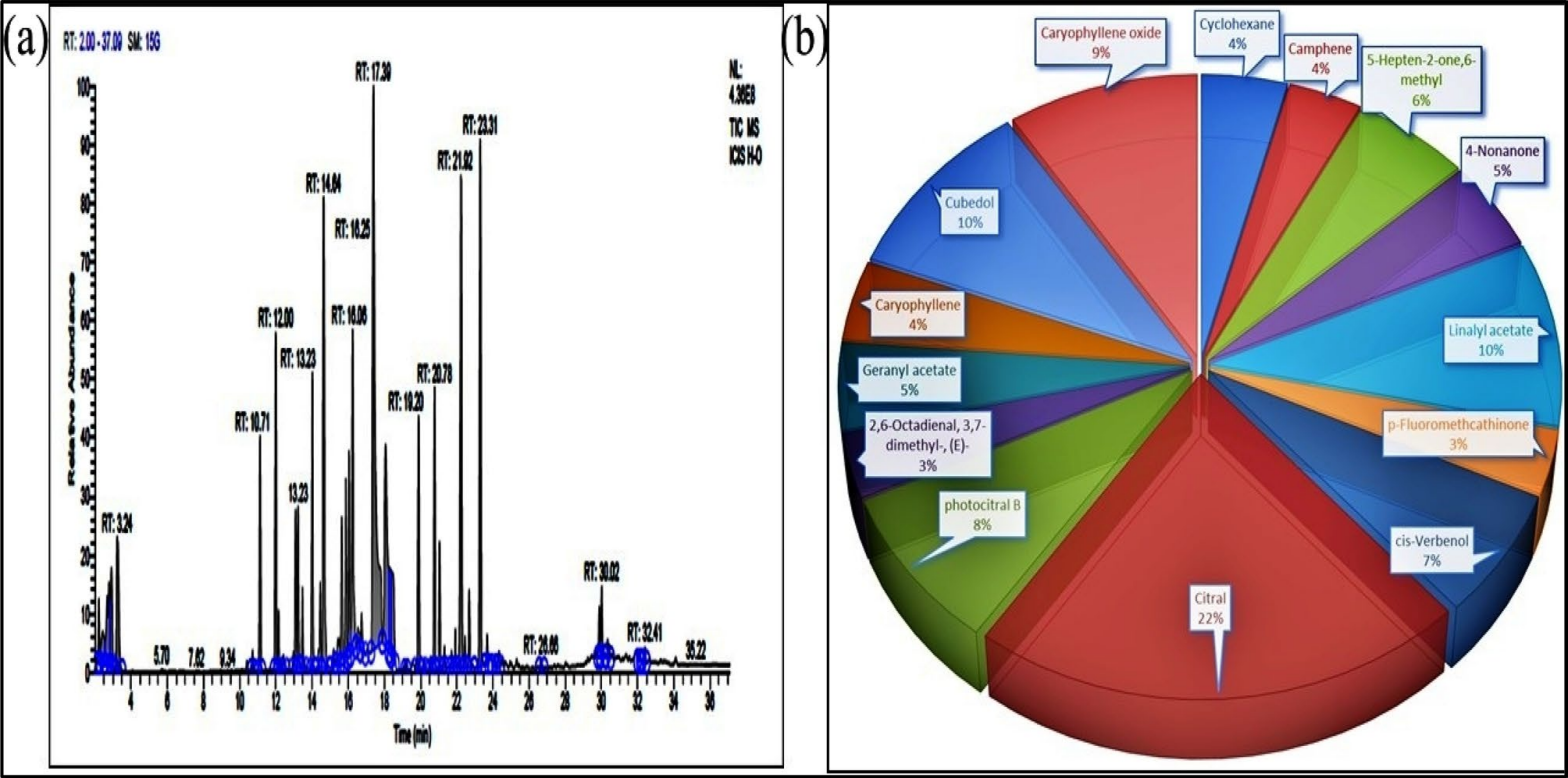
(**a**) Total ion chromatograms of GC–MS, (**b**) Percentage chemical composition of essential oil of *C. flexuosus* from site II—Haryana (S2).

In S2, chromatogram of essential oil of *C. flexuosus* were characterized with 49 prominent peaks at different retention times 2.22–32.41, etc. Citral and Cubedol were the major compounds observed at RT-17.39 with a peak area of 15.19% and RT- 22.24 with 7.09%, respectively. Other compounds observed were Linalyl acetate, Caryophyllene oxide, photocitral B, cis-Verbenol, 4-Nonanone, Cyclohexane, Camphene, Geranyl acetate and Caryophyllene, (Fig. 2). Other remaining minor identified compounds observed were octanal, α-Pinene, Acetyl eugenol, Hinesol, tau-Murolol, Isocaryophillene and Camphorsulfonic acid.

### From site III—Dehradun (S3)

**Figure 3:**
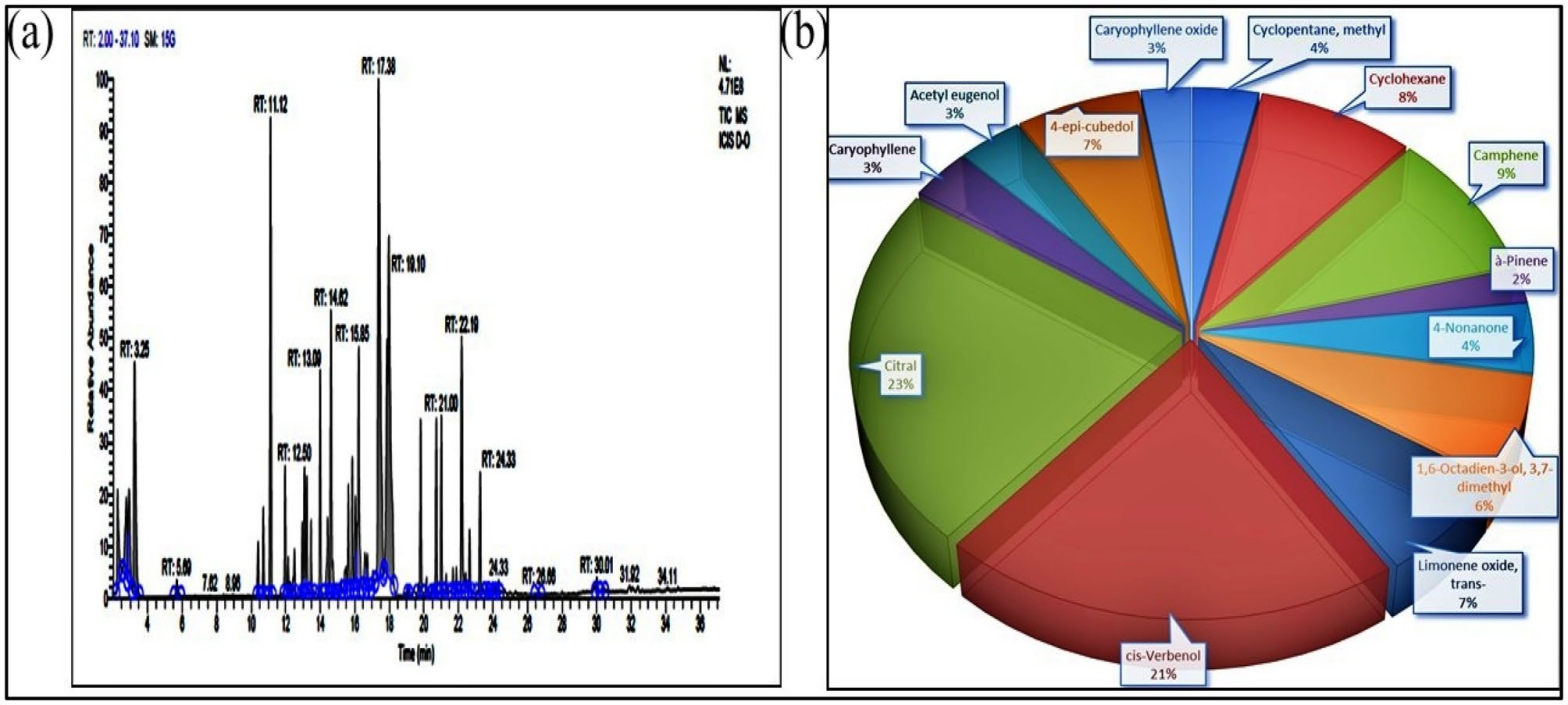
(**a**) Total ion chromatograms of GC–MS, (**b**) Percentage chemical composition of essential oil of *C. flexuosus* from site III—Dehradun (S3).

The S3 chromatogram of essential oil was characterized with 47 prominent peaks at different retention times from 2.26 to 30.01. Compounds Citral observed at retention time 17.97 with peak area 16.14%, and cis-Verbenol observed at RT-17.38 with peak area 14.92% respectively were significant constituents. Other components observed were Camphene, Cyclohexane, 4-epi-cubedol, Limonene oxide, 4-Nonanone, cyclopentane; methyl, Caryophyllene, Acetyl eugenol and Pentane, 2-methyl (Fig. 3). The remaining minor identified compounds are d-limonene, Caryophyllene oxide, α-Pinene, Humulene, Cubedol, Decanal.

Compounds such as hydrocarbons, terpenes, esters, alcohols, mainly aldehydes, and ketones have constantly been registered in the literature^39^. Lemongrass essential oil contains active ingredients like citral, an aldehyde; myrecene, an alkene; citronellol and geraniol are alcohols. The biological potency of essential oil procured from *Cymbopogon* species has been reported to be due to the presence of Citral^40^. The quantity of compound Citral was observed highest in S3, i.e., 16.14%, whereas, in S2 it is 15.19% and in S1 it is 13.61%, respectively. Although the chromatograms of all the 3 sites were unique, but some common constituents were found in all sites. The major common compounds were Citral, Caryophyllene oxide, cubedol, α-ocimene, 4-Nonanone, Caryophyllene, Geranyl acetate, photocitral B, linalyl acetate, α-pinene, d-limonene, cyclohexane, cis-Verbenol, (Pentane,2-methyl), Cyclopentane that was found in all the sites having different altitudes^41^. The results are consistent with the existed information. Moreover, the presence and concentration (amount) of chemical constituents varied, and that was due to the involvement of diverse features, such as climate and altitude as shown in Table 1.

**Table 1:** Altitudinal disparity in chemical constituents of essential oil of *Cymbopogon flexuosus*.

Site-II	Compounds name	Area%		
		Site-I	Site-II	Site-III
1	Citral	13.61	15.19	16.14
2	Caryophyllene oxide	2.36	6.64	1.85
3	Cubedol		7.09	0.35
4	α-Pinene	12.39	1.79	1.55
5	4-Nonanone	3.20	3.43	3.08
6	Caryophyllene	1.89	3.04	2.28
7	Geranyl acetate	6.34	3.29	–
8	Photocitral B	10.79	5.87	6.35
9	Linalyl acetate	7.72	6.95	4.75
10	α-ocimene	0.36	0.96	1.03
11	d-Limonene	5.52	1.93	1.75
12	Cyclohexane	3.10	2.99	5.87
13	Cis-verbenol	2.42	4.71	14.92
14	Pentane, 2-methyl	0.55	0.71	2.27
15	Cyclopentane, methyl	2.33	2.42	2.48

### Chemical and functional evaluation of AuNPs

**Figure 4:**
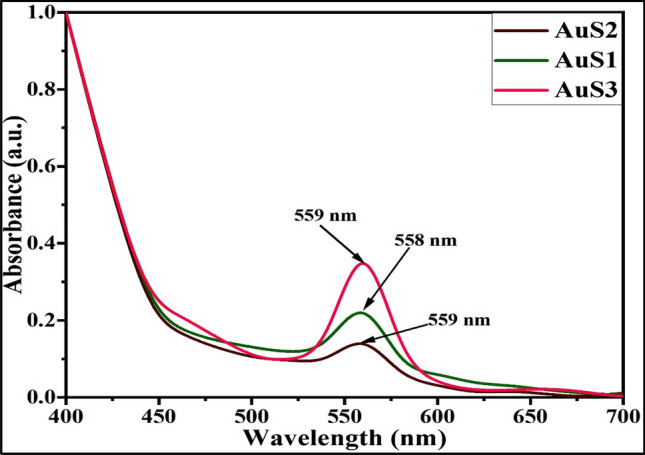
UV–Vis spectra of AuNPs using essential oil extracts of *C*. *flexuosus* from different altitudes, i.e., Palampur AuS1 (**a**), Haryana AuS2 (**b**), and Dehradun AuS3 (**c**).

The formation and chemical structure of biosynthesized AuNPs has been evaluated using UV–Vis and FTIR spectroscopies. UV–Vis spectroscopy identifies the formation, stability, size, and band gap of metallic nanoparticles. The introduction of Au particles to surface plasmon resonance generates a vibrant color in a colloidal solution, which signifies the formation of Au NPs. The bioreduction process converting Au^3+^ ions into AuNPs is characterized by visual color alteration from light yellow to wine red, as reported in the literature^42^. Similar observations of visual annotation have been observed during the reaction. Furthermore, SPR results in a high absorption band, which is measured by employing a UV–Vis spectrophotometer. The UV–Vis spectrum for lemongrass essential oil mediated gold nanoparticles and shown in Fig. 4. The color change was further established by the UV–Vis spectra obtained for reaction solution, which showed UV–Vis spectra peaks of AuS1, AuS2, and AuS3 at 558, 559, and 559 nm, respectively. It is consistent with earlier reports on manufacturing gold particles by utilizing essential oil^43^. It is qualitative evidence that bioreduction of trivalent Au ions using lemongrass essential oil is suitable to produce AuNPs. These outcomes conform with the findings of Suman et al. who determined that the biofabrication of AuNPs from *Morinda citrifolia* aqueous root extract unveils a peak of absorbance at 540 nm^44^. Analogous information has also been acknowledged by Sujitha and Kannan (2013), who analyzed that the biofabrication of AuNPs utilizing citrus fruits exhibited the peak of absorbance at 550 nm^45^.

**Figure 5:**
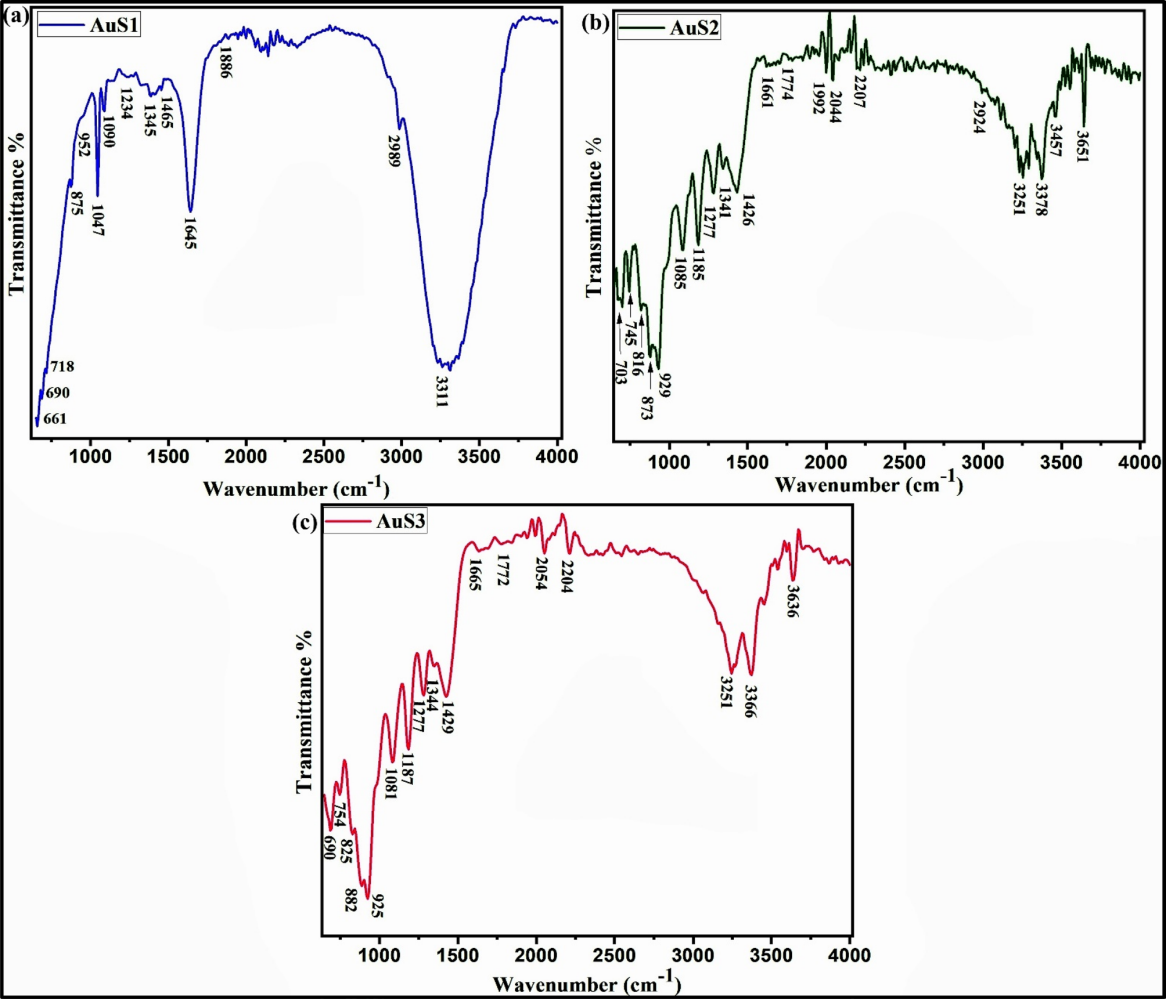
FTIR spectra for AuNPs using essential oil of *C*. *flexuosus* from different altitudes of Palampur (AuS1) (**a**), Haryana (AuS2) (**b**), and Dehradun (AuS3) (**c**).

Furthermore, FTIR spectra revealed the presence of alkene, primary amide, amine, aldehyde, alcohols, ketone, and ethers in prepared samples (Fig. 5). These functional groups identified are in agreement with compounds reported in the literature and the ones identified in GC–MS analysis. For instance, alkenes (citronellene, caryophyllene, a-pinene, myrcene), aldehyde (Citral), alcohols (cis-Verbenol, geraniol), and ketone (4-Nonanone) were present^46^. To support the results of GCMS and synthesized nanoparticles, additional studies were conceded by FT-IR investigation to be familiar with the complex configuration and interaction of prominent functional groups incorporated in essential oil encapsulation. These outcomes assisted us in validating the existence of essential oil in synthesized nanoparticles. The characteristic peaks on 3311 cm^−1^, 3251 cm^−1^, 3636 cm^−1^ and 3457 cm^−1^ indicate the presence of hydroxyl and amino groups, 1429 cm^−1^, 1426 cm^−1^ and 1465 cm^−1^ indicates the involvement of carboxylate group and the peak at 1080 cm^−1^, 1090 cm^−1^, 1081 cm^−1^, 1047 cm^−1^, 952 cm^−1^ indicates C–O stretching. The C–N stretching peaks were observed at 1185 cm^−1^, 1187 cm^−1^, 1085 cm^−1^.

Moreover, there are several minor and a few significant peaks produced due to the existence of essential oil. The peaks between 1750 and 1650 cm^−1^ represent the occurrence of citral in the FTIR of essential oil^47^. It is clear that characteristic peaks that occurred in the essential oil spectrum are important in the essential oil mediated AuNPs. The peaks at 1665 cm^−1^, 1661 cm^−1^, and 1645 cm^−1^ demonstrate Citral's existence, an extremely remedial component in lemongrass essential oil. The reallocate in the peaks determines the probable relations and thriving encapsulation of oil hooked on the carrier. Prominent peaks 3457 cm^−1^, 2924 cm^−1^, 1664 cm^−1^, and 1085 cm^−1^ into FTIR of essential oil mediated nanoparticles indicate corresponding N–H, O–H, C=O, and CH-OH functional groups. Hence, the FTIR outcome represents that the essential oil has a strong ability to form metal nanoparticles through the antioxidant effect of essential oil. Therefore, structural analysis strongly anticipates essential oil's role as stabilizing and reducing agents for AuNPs fabrication.

### Morphological and structural evaluation of AuNPs

The morphology, ordering, and crystal structure of biosynthesized AuNPs has been evaluated using X-ray diffraction spectroscopy, Scanning electron, and transmission electron microscopy.

**Figure 6:**
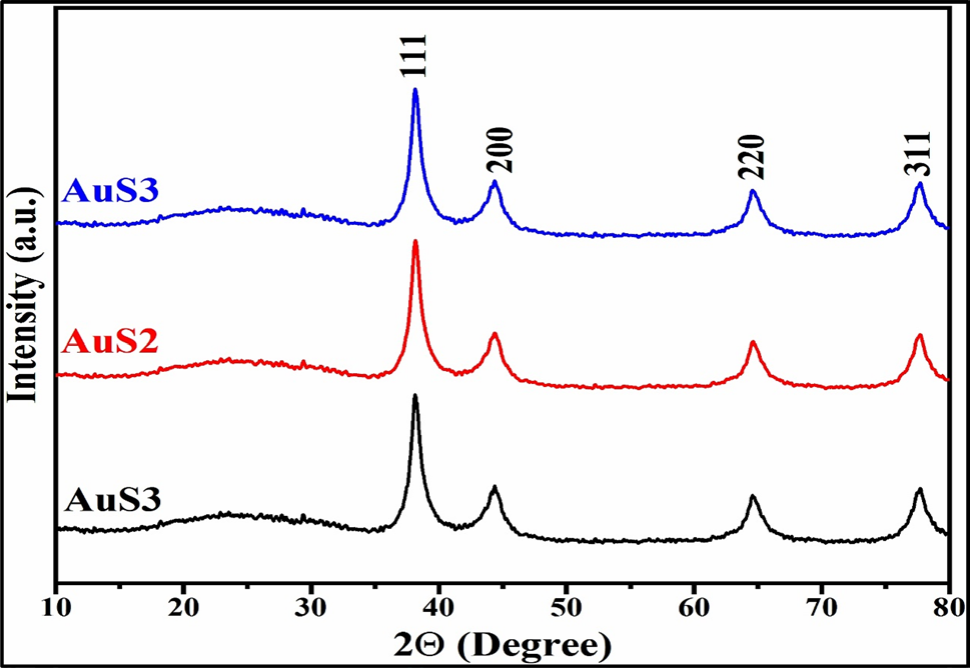
X-ray Diffraction for AuNPs using essential oil of *C*. *flexuosus* from different altitudes of Palampur (AuS1) (a), Haryana (AuS2) (b) and Dehradun (AuS3) (c).

The crystallographic arrangement, grain size, and ordering of essential oil-mediated AuNPs are evaluated by the X-ray diffraction (XRD) technique^45^. The diffraction peaks lying on 2θ were 38.22, 44.52, 64.54, and 77.69 (Fig. 6), which should be consequently allocated to crystalline planes (111), (200), (220), and (311) respectively showed the Face centered cubic configuration of gold nanoparticles (JCPDS 04-0784)^48^. The average range of crystallite size is estimated between 4 and 11 nm. It is noticeable that the intensity of (111) dominates, indicating that particles are primarily oriented in (111) directions. The current outcomes are inconsistency with the conclusion of Suman et al. and Boruah et al.^44,49^.

**Figure 7:**
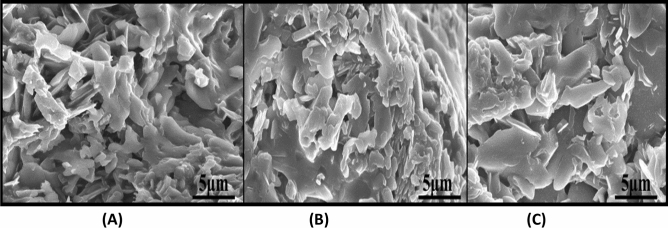
(**A**–**C**) SEM micrographs of AuNPs synthesized from the essential oil of *C. flexuosus* from diverse altitudes of (**A**) Palampur (AuS1), (**B**) Haryana (AuS2), and (**C**) Dehradun (AuS3).

**Figure 8:**
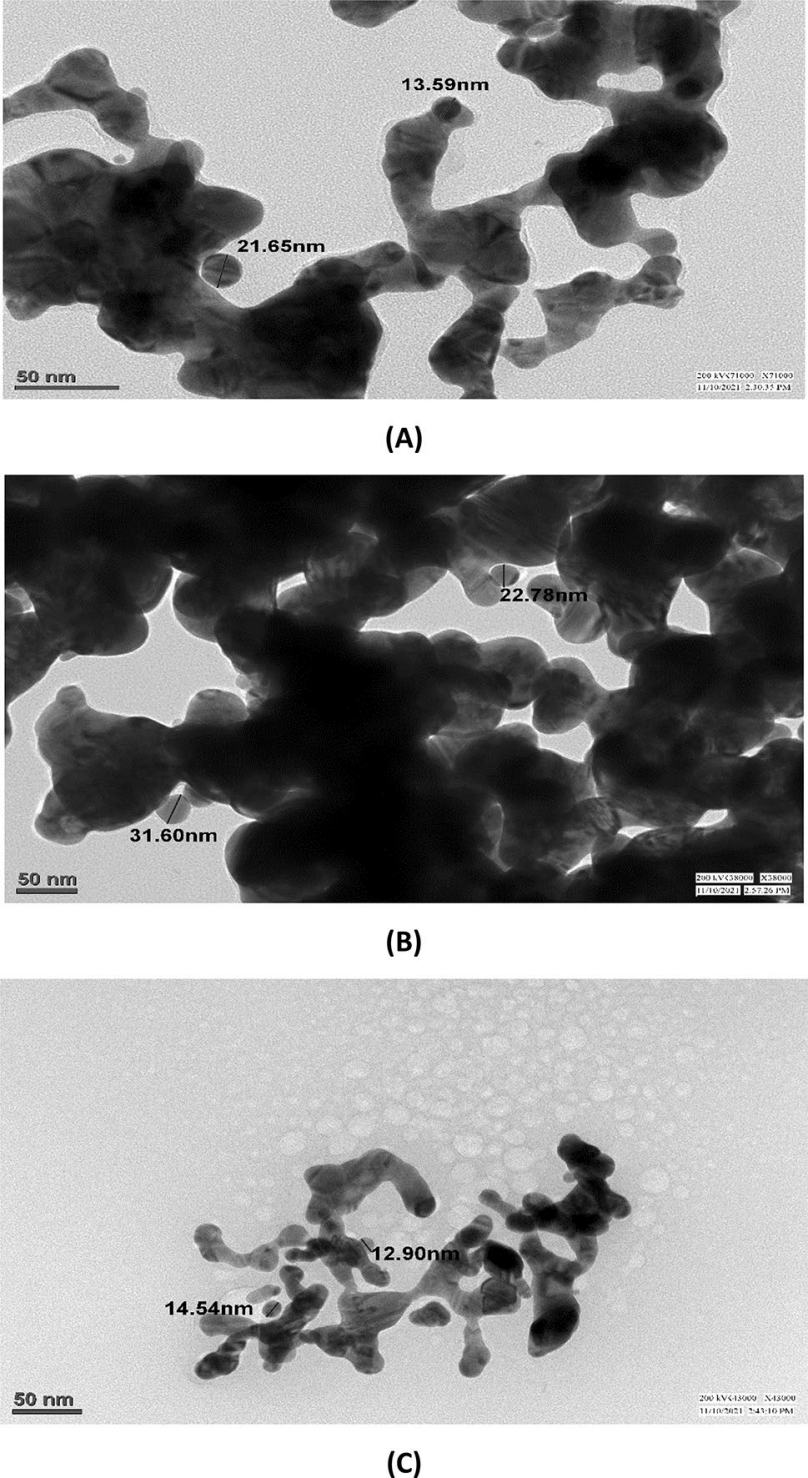
(**A**–**C**) TEM micrographs of AuNPs synthesized from the essential oil of *C. flexuosus* from diverse altitudes of (**A**) Palampur (AuS1), (**B**) Haryana (AuS2) and (**C**) Dehradun (AuS3).

Further, SEM analysis results demonstrated that the essential oil-mediated AuNPs are in irregular nano-disc shape with diffused morphology, as shown in Fig. 7. It is observed that grain size varies for all three samples. It is attributed to the variation in chemical constituents for different extracts. It is a well-known fact that essential oil or the green extract acts are responsible for the surface modification, and hence the variation in grain size is observed. It is also observed that grains are highly agglomerated, and the prediction of precise morphology and particle size of the prepared sample requires other substantial results, Such as TEM micrographs (Fig. 8). The reason for high agglomeration with diffused morphology can be ascribed to the presence of essential oil in the synthesis, which is following previously reported literature. Hence, SEM results strongly anticipate the existence of essential oil in prepared AuNPs.

Hence, the morphology and particle size of prepared samples are evaluated using the TEM technique. TEM micrographs reveal smaller crystallites' self-assembly to form the branched structure, as shown in Fig. 8A–C. The average crystallite size ranges from 10 to 32 nm, which is insecure conformity with the XRD investigation. However, a slight variation is observed between TEM and XRD examinations. This is for the reason that in TEM micrographs, we openly envision the irregularities, such as dissimilarity in shape or size of the particles, while in XRD, the Scherrer technique is engaged to estimate the particles based on the hypothesis that the particles are spherical in shape. Hence, the electron microscopies and XRD analysis reveal AuNPs with an average size below 50 nm. Such nanosized particles possess high surface-to-volume ratios, which anticipate their potential candidature in surface phenomenon-based applications such as photocatalytic activities and antimicrobial behavior.

### Evaluation of photocatalytic efficacy of AuNPs

**Figure 9:**
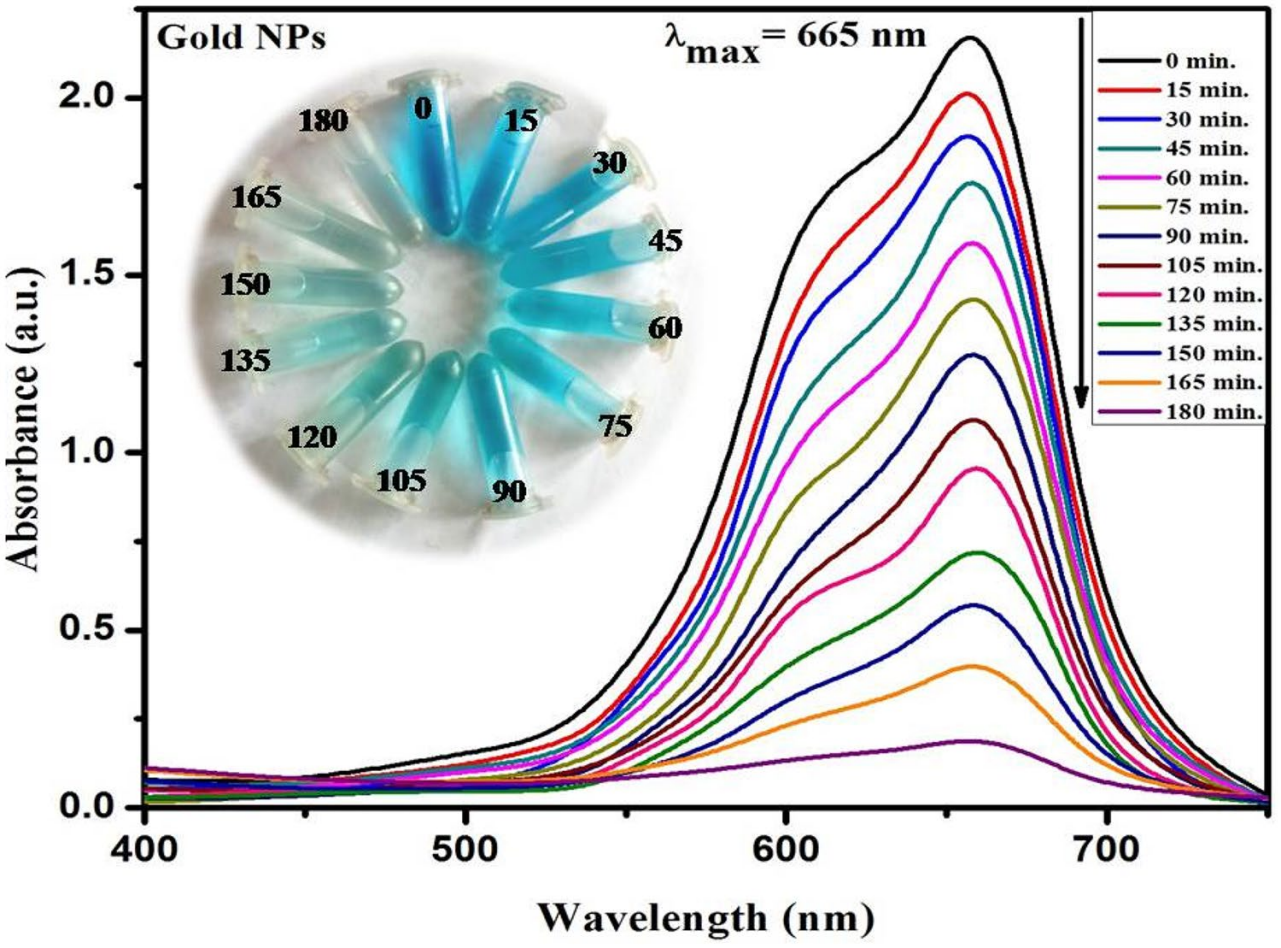
UV–Vis absorption spectra of methylene blue dye using AuNPs.

**Figure 10:**
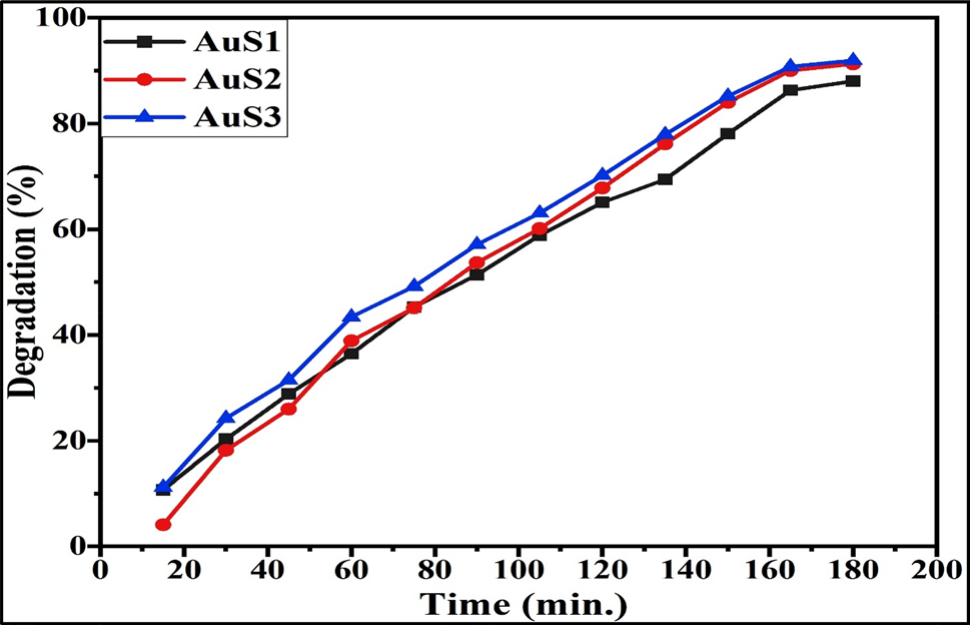
Photodegradation percentage of MB using gold nanoparticles fabricated from essential oil of *C. flexuosus* from different altitudes.

The decrease in peak absorbance of methylene blue at 665 nm as a purpose of contamination time depicts the photocatalytic assessments of applied gold nanoparticles (Fig. 9). The *Cymbopogon flexuosus* essential oil mediated AuNPs has shown remarkable photodegradation activity against methylene blue contaminated water sample at various degradation intervals. A considerable decent in absorption intensity of MB was observed within the initial 15 min, which ultimately disappeared entirely within 180 min. Figure 10 illustrates the time outline of MB dye photodegradation efficacy. Within 60 min of photocatalysis, approximately 43.3% in S3 (which is highest among all the sites), 36.4% in S1, and 38.9% in S2 methylene blue dye concentration was photodegraded under visible sunlight irradiation, yielding an ending MB degradation efficacy of 91.8% in S3 (maximum), 87.9% in S1 and 91.2% in S2 within 180 min under sunlight. In the early stage of photodegradation, the accessibility of numerous photocatalytic sites and a high absorption slope encouraged the fast color deterioration. The photodegradation of Methylene Blue (MB) confirms the amphoteric nature of AuNPs.

In the existence of sun light, the photodegradation ability of gold nanoparticles proposed a three-step mechanism, which involves the generation and transfer of electron–hole pairs, radical generation and then degradation of dye. The electron and hole pair from gold conduction and valence bands are generated in the first step by irradiating the material with sunlight, as explained in Eq. (1).

1 $${\text{Au}} + h\nu \to {\text{Au}} \left( {e_{cb}^{ - } + h_{vb}^{ + } } \right)$$

The rate of recombination of charge carriers is decreases with sunlight irradiation and facilitating electron–hole pair separation on the surface. In the second phase, oxygen molecule interacts with surface electrons to generate superoxides, which are then converted to peroxide molecules. In addition, the surface holes oxidize the water molecules, resulting in the formation of hydroxyl ions as shown in Eqs. (2)–(4) ^50^.

2 $${\text{H}}_{2} {\text{O}}_{2} + e^{ - } \to \cdot {\text{O}}_{2}^{ - } + 2{\text{H}}^{ + }$$

3 $${\text{H}}_{2} {\text{O}}_{2} + \cdot {\text{O}}_{2}^{ - } \to \cdot {\text{OH}} + {\text{OH}}^{ - }$$

4 $${\text{H}}_{VB}^{ + } + {\text{H}}_{2} {\text{O}}\left( {{\text{OH}}^{ - } } \right) \to {\text{OH}}^{ \cdot } + {\text{H}}^{ + }$$

The above formed intermediates are exceedingly unbalanced, and when they act in response with the dye substituent, leading its breakdown to mineralized yields as presented in Eq. (5) ^51^.

5 $${\text{MB}}\;{\text{ Dye}}\;{\text{ molecule }} + \, \left( {{\text{OH}}^{ \circ } ,{\text{ HOO}}^{ \circ } ,{\text{ H}}^{ + } {\text{or H}}_{{2}} {\text{O}}_{{2}} } \right) \to {\text{Degraded/mineralized}}\;{\text{ products}}.$$

A large effective surface area was provided by the nanosized essential oil mediated AuNPs to facilitate electron–hole pair separation for distinct radical species that readily helps to disintegrate the dye molecule. The findings of photodegradation investigations revealed that as-fabricated gold nanoparticles have significant photocatalytic activity (Table 2). The almost total disintegration of methylene blue, with around 91.8% photodegradation within 3 h, indicates that as-prepared gold nanoparticles had substantial photocatalytic efficiency. As shown in Table 3, crystallite size decreased photocatalytic efficiency increased. So photocatalytic activity depends on the crystallite of nanoparticles also.

**Table 2:** Comparative study of gold nanoparticles for photocatalytic performance against MB dye.

Nanoparticles	Method	Dye	Time (min)	Degradation %	Ref.
Au Nps on ZnO Nps	Sol–gel method	MB	180	82.1	^52^
Au Nps	Green synthesis	MB	360	84	^53^
Au Nps	Green synthesis	MB	420	76.12	^54^
Au Nps	Green synthesis	MB	300	50	^55^
AuS1, AuS2, and AuS3 Nps	Green synthesis	MB	180	87.9, 91.2, and 91.8	Current work

**Table 3:** In terms of physicochemical properties, sunlight-assisted photocatalytic performance of produced Au NPs.

Samples	Crystallite size (TEM)	Crystallite size (XRD)	Photo-catalytic efficiency (%)
AuS1	7–30 nm	7.1 nm	87.9
AuS2	8–35 nm	6.25 nm	91.2
AuS3	5–20 nm	6.40 nm	91.8

### Assessment of antibacterial performance of AuNPs

**Figure 11:**
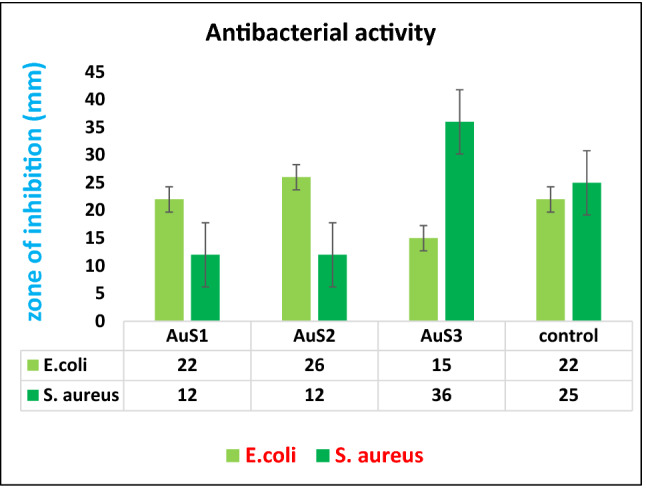
Zone of inhibition of selected bacteria by synthesized gold nanoparticle.

The antimicrobial potential of AuNPs and *Cymbopogon flexuosus* essential oil was analyzed adjacent to diverse multi-drug unwilling microorganisms. The results presented in Fig. 11 confirmed the antimicrobial efficiency of essential oil and AuNPs against several famous pathogenic indicator strains. The essential oil demonstrates powerful antibacterial efficacy together against gram positive and negative bacteria. Biofabricated AuNPs in the current investigation also revealed remarkable antibacterial efficiency as a Biocidal agent, and this may be owed to the smaller size of AuNPs, enlarged surface area and chemical structure. The dissimilar nanosized AuNPs may be effortlessly taken up by the microbial cells. Similarly, nanoparticles displayed a much profound consequence on all preferred microorganisms. Lemongrass essential oil has citral as active compound which is known for its biocidal activities. Therefore, loading of essential oil was suited for target drug delivery applications.

Antibacterial activity of biofabricated AuNPs from essential oil of *C. flexuosus* from various elevations have been examined against two infectious bacteria, i.e., *S. aureus* and *E. coli* using well diffusion assay and zone of inhibition as represented in Fig. 11. Wells were loaded with the same concentration of synthesized nanoparticles from different altitudes, i.e., 80 µl. Maximum zone of inhibition (36 mm) have been observed with *S. aureus* from AuNPs synthesized from essential oil extract of AuS3 (Dehradun). Beside *E. coli* maximum inhibition zone (26 mm) is detected as of gold nanoparticles synthesized from essential oil of AuS1 (Palampur) and minimum zone of inhibition (22 mm) have been observed in AuS3 respectively. Comparative study of Au Nps for antibacterial efficiency is done in Table 4. The present outcomes corroborate with the findings of Abalaka et al*.* who investigated that *Pseudomonas pneumonia, Salmonella typhi* and *Staphylococcus aureus* were prone to fabricated AuNPs from *Prunus amygdalus* and *Gomphren acelosioides* leaf extracts^56^.

**Table 4:** Comparative study of gold nanoparticles for antimicrobial efficacy against *E. coli and S. aureus.*

Nanoparticles	Fabrication method	Zone of inhibition (mm)		Ref.
		*S. aureus*	*E. coli*	
Au Nps	Green synthesis	15 mm	16 mm	^57^
Au Nps	Green synthesis	9 mm	13.5 mm	^58^
Au Nps	Green synthesis	19.7 mm	17.7 mm	^59^
Au Nps	Green synthesis	10.6 mm	11.3 mm	^60^
Au Nps	Green synthesis	16 mm	11 mm	^61^
Au Nps	Green synthesis	13.33 mm	13.33 mm	^62^
AuS1, AuS2, and AuS3	Green synthesis	12, 12, and 36 mm	22, 26, and 15 mm	Present work

### Assessment of antifungal behavior of AuNPs

**Figure 12:**
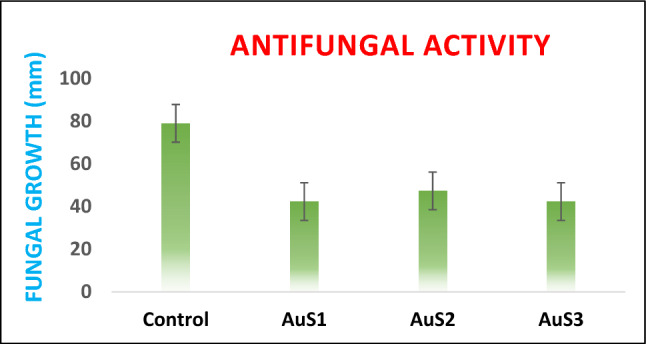
Fungal growth of selected fungal strains by synthesized gold nanoparticle.

In this study, fabricated AuNPs using essential oil of *C. flexuosus* were tested against *Fusarium oxysporum* employing well diffusion technique to evaluate their capability as an impending applicant for an antifungal negotiator. Antifungal activity of AuNPs was mostly comparable to the standard reference antifungal drug streptomycin to some extent. The amount of the antifungal representative was analyzed, depends on the inhibition zone size created throughout each well full of a suitable test sample. AuNPs synthesized from *C. flexuosus* essential oil showed notable antifungal activity against *Fusarium oxysporum.* The *F. oxysporum* strains expansion rate in the presence of the examined nanoparticles is obtainable in Fig. 12. Gold nanoparticles from site-I and site-III, i.e., AuS1 and AuS3 were highly active against *F. oxysporum*, they showed 46.41% of inhibition in both sites whereas, in site-II, i.e., AuS2 percentage of inhibition was 40.08%. Control growth was 79 mm at fullest. Comparative study of Au Nps for antifungal efficiency is done in Table 5. AuNPs were shown to be substantially more efficient against the development of harmful fungus (*F. oxysporum*) according to all observational and quantitative studies.

**Table 5:** Comparitive study of gold nanoparticles for antifungal efficiency against *Fusarium oxysporum.*

Nanoparticles	Fabrication method	Fungal growth (mm)	Ref.
Au Nps	Green synthesis	8.66	^62^
Au Nps	Green synthesis	14.2	^63^
Au Nps	Green synthesis	10	^64^
Au Nps	Green synthesis	12	^65^
AuS1, AuS2, and AuS3	Green synthesis	42.33, 47.33, and 42.33	Present work

## Conclusion

In summary, *Cymbopogon flexuosus* plant essential oil was utilized as a reducing, capping, and stabilizing agent for the fabrication of biogenic AuNPs of remarkable physicochemical, antibacterial, antifungal, and photocatalytic properties. The well characterized AuNPs of size ranging between 10 and 32 nm are suitable for diversified application to adopted green technology supported by plant extracted essential oil, pure water, and nearly harmless chemicals. The altitudinal disparity in phytochemical composition of essential oil has influenced the physicochemical, antimicrobial, and photocatalytic properties of fabricated AuNPs. The fabricated AuNPs exhibited excellent sunlight assisted photodegradation efficiency of around 91.8% against methylene blue dye within 3 h. Moreover, the superior antimicrobial properties of AuNPS compared to traditional medicinal remedies are attributed to presence of bioactive functionalities on surface of AuNPs as revealed from FTIR outcomes. These in vitro results demonstrate the promising therapeutic prospective of essential-oil mediated AuNPs in nanomedicines and wide range of biotechnological implementations.

## Experimental

### Materials and methods

Fresh leaves of *C. flexuosus* were collected from 3 different sites, i.e., site-I (Palampur), site-II (Haryana), site-III (Dehradun). The specimen was deposited in the Herbarium library of Shoolini University, Solan (H.P) under the Voucher No.-SUBMS/BOT-4798. Chloroauric acid (HAuCl_4_·H_2_O), Methylene Blue (MB), and acetone were procured from Loba Chemie. 150 g of fresh leaves of *Cymbopogon flexuosus* were hydro-distilled in a Clevenger apparatus in 1500 ml of de-mineralized water at 60 °C for 3 h, yielding 2 ml essential oil from site-I, 2.5 ml from site-II, and 4 ml, essential oil from site-III (yielding 1.3%, 1.6%, and 2.6%, respectively). The essential oil thus obtained is characterized through pale yellow color with a strong lemon-like odor and utilized for the NPS fabrication.

### Gas chromatography-mass spectrophotometric analysis (GC/MS) of the essential oil

GC (gas-chromatography) study was conceded employing Agilent technology chromatograph by HP-5 column (30 m × 0.32 mm, internal diameter 0.25 µm). Oven temperature is 60 °C to 210 °C at 3 °C/min withhold time of 8.5 min; injector temperature was 280 °C and temperature of the detector was 290 °C. The carrier gas was ${N}_{2}$ (1 ml/min) with 1:50 split ratio. The HP-5 MS capillary column was used by Helium as the carrier gas at 1 ml/min. Oven temperature of GC was planned from 60 to 210 °C at a pace of 3 °C/min and afterward amplified from 210 to 240 °C at a rate of 20 °C/min and then set aside firm at 240 °C for 8 min. The injector temperature was 280 °C. Then MS was scanned above 40–550 amu with 70 eV of an ionizing voltage. The retention index was analyzed using retention times of n-alkanes (${C}_{8}$–${C}_{25}$) injected following the extracted material of the plant beneath similar chromatographic circumstances. For all components, retention indices were resolute according to the technique that utilizes n-alkanes as standard, and their retention times were determined^66^. The components were recognized with the association of retention index among those present in the literature in the instrument library.

### Green synthesis of biogenic AuNPs

For bio-fabrication of AuNPs, aromatic oil was diluted using acetone (2 ml of essential oil in 10 ml of acetone). 7 ml of adulterated oil with vigorous stirring was added to 0.1 M concentration of chloroauric acid (by dissolving 0.339 g in 10 ml distilled water) at 40–60 ℃ temperature for about 2 h on the magnetic stirrer^67^. 1.5 ml of 0.5 M NaOH was added to maintain pH. The yellow color was observed to change with wine red which specified the enhanced formation of AuNPs. At different intervals, the solution was sampled, and UV–Vis spectrophotometer (Thermo Fisher Scientific Evolution 160 UV–Vis spectrometer) was used to monitor surface plasmon resonance at various time points in the solution. During the reaction, the color was observed to change gradually. The mixture was kept stable for 2 h, and precipitates were allowed to settle down. Then precipitates were centrifuged at 5000 rpm for 15 min and 4–5 times washed with distilled water. The final samples were dried at 40 °C to obtain AuNPs.

### Characterization tools for evaluation of physicochemical properties of AuNPs

AuNPs were characterized to determine their properties and potential uses in engineering and science applications using various characterization techniques. Absorbance peaks (UV–Vis spectrophotometer) were used to monitor and characterize AuNP's appearance and stability. Scanning the reaction mixture with a Lambda Perkin Elmer UV–Vis spectrophotometer in the 400–700 nm series allowed us to track the progress of the reaction. Morphological and particles dimension analysis was finished through Scanning Electron Microscope (SEM) nova nano SEM 450 and TEM investigation (Transmission Electron Microscopy). XRD was performed to figure out the crystal structure of AuNPs (X'Pert PRO X-ray diffractometer, PAN analytical). The Fourier Transform Infrared Spectrophotometer (FTIR) Perkin Elma within the range of 650 cm^−1^ to 4000 cm^−1^ was used to identify functional groups involved in the reduction reaction and stability of Au NPs. Samples were centrifuged and dried up before performing FTIR analysis.

### Photocatalytic activity estimation

AuNPs were utilized as probe catalysts to examine the photodegradation of methylene blue dye in synthetic wastewater treatment in the presence of sunlight. The experimental methodology includes the dispersion of 100 mg of AuNPs in 200 ml of dye tainted wastewater at a concentration of 10 ppm. The solution was continuously stirred for 30 min at 100 rpm under darkness to attain adsorption–desorption constancy with dye and nanoparticles. The equilibrated dye and nanoparticles solution was afterward subjected to sunlight to observe the photocatalysis of AuNPs towards dye degradation. The reaction mixture was withdrawn after 15 min interval for dye concentration evolution. To separate the nanoparticles from the rest of the sample, they were centrifuged at 12,000 rpm, and, finally, the resulting floatable was exposed to adsorption measures using a UV spectrophotometer. In the meantime, the dye absorbance decreased (*λ*max = 665 nm) gradually provided the decolorization rate as well as photocatalytic efficiency of AuNPs as calculated with Eq. (6) ^68^.

6 $$\eta \, = \, \left[ {\left( {{\text{A}}_{0} {-}{\text{ A}}_{{\text{t}}} } \right)/{\text{ A}}_{0} } \right] \, \times { 1}00$$

where A_0_ and A_t_ are primary and final absorbance after a specific reaction time, respectively.

### Antibacterial assay to evaluate the antibacterial efficiency

Well diffusion assays were used to investigate the antibacterial efficiency of biosynthesized AuNPs against pathogenic bacteria^69^. *S. aureus* (a Gram-positive organism) and *Escherichia coli* were used as the test organisms (Gram-negative). The media used to cultivate the bacterial strains was Nutrient Agar. Nanoparticles synthesized from *C. flexuosus* essential oil and tested against the bacterial culture at various concentrations were examined. The Nutrient Agar agar plates with strains swabbed and Whatman No. 1 filter paper were used to prepare wells (9 mm diameter). On the plates, bacterial culture was grown, and wells were made to which 80 μl of the sample was taken from 25 mg/ml stock solution. For the comparison, Ampicillin was taken as a positive control for antibacterial activity, while DMSO was used as a negative control. It took 24 h to incubate the plates at 37 °C. The antibacterial effectiveness of the samples was assessed using a gauge to their inhibition zone diameter, which has a millimeter diameter.

### Antifungal activity

Bio-fabricated AuNPs were examined for antifungal assessment against pathogenic fungus using a well diffusion assay. The fungus used was *Fusarium oxysporum.* For the fungal strain PDB was used as the culture medium. Different concentrations of *C. flexuosus* essential oil-derived nanoparticles from various altitudes were tested against fungal strains. 6 mm of the fungal disc was taken. Wells were made in Potato Dextrose Agar (PDA) media. Plates were cultured, and wells were made to which 60 μl of the sample was taken from 5 mg/ml stock solution. For comparison, Streptomycin was taken as a positive control. For 6 days, the plates were incubated at a temperature of 27°^69^. The antifungal effect of the samples was assessed using a gauge to their inhibition zone diameter.

## Data Availability

The datasets used and/or analysed during the current study available from the corresponding author on reasonable request.
